# An ingenious design from nature to accelerate the repair of long-bone critical defects: the longitudinal tubular transverse interconnection structure of deer antlers

**DOI:** 10.1016/j.mtbio.2025.102090

**Published:** 2025-07-15

**Authors:** Chenyu Wang, Wenbo Yang, Lanfeng Song, Lanqing Cao, Guokun Zhang, Xiaofan Gao, Xiujie Zhu, Shipu Jia, Xiang Yue, Chunyi Li, Jincheng Wang, Xin Zhao, Haotian Bai

**Affiliations:** a Department of Plastic Surgery, The First Hospital of Jilin University, China; b Department of Orthopedics, The Second Hospital of Jilin University, China; c Department of Pathology, The Second Hospital of Jilin University, China; d Institute of Antler Science and Product Technology, Changchun Sci-Tech University, China

**Keywords:** Velvet antler, Anisotropic structure, Critical bone defects, Osteoconduction, Decellularized matrix

## Abstract

Deer antlers, the only mammalian bony organs capable of complete regeneration, exhibit a growth rate of 2.7 cm/day, far surpassing human long bones (1 mm/day). Long-bone critical defects (LBCDs) occur when defects exceed intrinsic healing capacity. While antler stem cells drive regeneration, their immunogenicity limits clinical translation. Antler extracellular matrix (ECM) components have been proven to enhance bone repair, the role of its unique "longitudinal tubule-transverse connection" structure remains unexplored. Here, matrix scaffolds (devoid of cellular/active components) were prepared along longitudinal (L) or horizontal (H) axes, with cancellous scaffolds (R) as controls. Histological and in vitro analyses confirmed structural integrity and immunogenicity elimination. Bone marrow mesenchymal stem cells (BMSCs) exhibited structural guidance in morphology and migration on L. Ectopic implantation revealed no intrinsic osteogenic activity but demonstrated robust alignment of soft tissues along scaffold scaffolds. In rat femoral segmental defect models, L induced significantly greater depth and volume of oriented new bone (vs. H or R) while effectively blocking fibrous encapsulation. This study identifies antler-specific structural topology—rather than cellular or biochemical factors—as the critical osteoconductive driver enabling rapid bone regeneration. The findings establish a proof-of-concept for bioinspired structural designs in addressing LBCDs, providing guidance for the development of antler-derived bone replacement implants and biomimetic design of additive manufacturing implants.

## Highlights

• Antler structure is an independent factor that promotes LBCDs repair.
• Antler antler has a highly anisotropic structure across scales.
• Antler antler structure can shield soft tissue invasion in the repair of LBCD.
• Antler antler structure can guide orderly bone regeneration in the repair of LBCD.

## Graphical abstract

**Figure:**
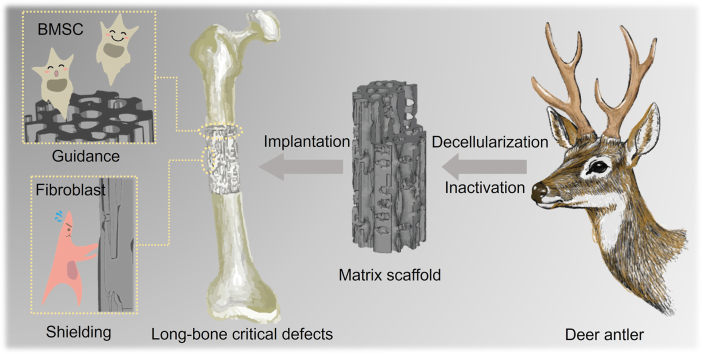

## 1 Introduction

Human bone is a tissue with some regenerative capacity. When a bone defect is very small, MSCs differentiate directly into osteoblasts and secrete extracellular matrices, which mainly contains collagen and calcium, to directly repair the defect [1]. When the bone defect is further enlarged, stem/progenitor cells aggregate and differentiate into chondrocytes to forma cartilaginous mass, accompanied by vascularization and calcification, which is eventually replaced by bone [2,3]. However, when the defect exceeds the self-healing capacity of the bone, completion of the repair has to rely on interventions, which are also known as critical bone defects. Long-bone critical defects (LBCDs) are defined as the bone loss in length longer than one and half of the long-bone diameter, or longer than one-fifth to one-fourth of the long-bone length. LBCDs have always been a great challenge in orthopaedics, which can be caused by congenital bone disease, arthritis, osteomyelitis, bone nonunion, bone infection, bone exposure, trauma, and bone tumour excision [4].

Autologous bone graft remains the gold standard for the treatment of LBCDs because autologous bone contains the three core elements needed for bone regeneration, i.e., cells, factors, and scaffold [[5], [6], [7]]. However, autogenous bone sources are often insufficient and face complications of bone defects in the donor area [8,9]. The Masquelet technique, an emerging alternative approach first described by Alain Masquelet, consists of a two-stage procedure that that can significantly reduce the amount of autologous bone needed [10]. In the first stage, the defect is temporarily filled with a polymethylmethacrylate (PMMA) spacer to create an induced membrane that can resist intrusion of the surrounding soft tissue. In the second stage of the procedure, the PMMA is removed and autogenous bone and bone substitute material are implanted within the induction membrane. The induction membrane does a good job of blocking soft tissue invasion into the area of the bone defect preventing resorption of the grafted bone, but it requires a second surgery and still does not prevent the use of autogenous bone [11]. For these reasons, bone replacement graft materials have received increasing attention in recent years.

Bone replacement graft materials include many types, such as allogeneic bone, xenograft bone, decalcified bone, bone decellularized matrix, etc [12,13]. Previous studies on bone replacement materials mostly focused on cells and bioactive factors, which were usually related to osteoinduction [14,15]. The term osteoinduction refers to the process of stimulating the differentiation of mesenchymal stem cells into osteoblasts in a non-skeletal environment by means of specific biomaterials or factors, which in turn promotes the formation of new bone [14,15]. Recently, more and more attention has been paid to the influence of structure on bone repair, which may be related to osteoconduction. Osteoconduction is the process by which biomaterials support the migration, attachment, proliferation and new bone formation of bone cells (e.g. osteoblasts) by providing a physical scaffold or surface structure [16,17]. It is essentially “passive guidance”, i.e., the material serves as a template to facilitate the gradual extension of bone tissue along its surface or pore space in an environment where there is already a margin of bone tissue [18,19]. For example, it is widely recognized that cancellous bone is more suitable for bone grafting than cortical bone, because cancellous bone contains a porous structure that allows new bone to grow into it, which is associated with osteoconduction, whereas cortical bone can only provide support [20].

More in-depth studies have found that structure has an important effect on osteoconduction [21,22]. For example, in the cancellous bone region, irregular randomized porous structures [23] that mimic bone trabeculae as well as triply periodic minimal surface (TPMS) structures [24] that mimic the natural curvature of bone trabeculae have been found to be advantageous in repairing cancellous bone. The pore structure of the scaffold affects cell penetration and inward growth of bone and blood vessels, ultimately determining the amount and direction of bone formation [16,17,23,24]. However, unlike cancellous bone, long bone has a specific anisotropic structure with the bone matrix oriented parallel to the bone axis, which may be related to its long-term weight bearing [25]. The cortical bone of the long bones is also not completely dense, containing longitudinal haversian tubes, which are tens of micrometers in diameter and connected transversely by Volkmann's canals, to maintain its nutrient supply and waste metabolism [26]. Therefore, special structures may be required for the repair of LCBD.

Similar to the same process of human bone development and repair, antler formation is an endochondral ossification process [27]. However, unlike human bone, antler can be regenerated at the point of cutting and is the only fully regenerable bony organ in mammal. During the rapid growth period of deer antlers, the growth rate can reach 2 cm/day [28], in contrast, the growth rate of human bone can only reach 1 mm/day at most [29]. In previous studies, our team identified an antler stem cell in antler velvet, which has an extremely strong proliferative capacity and osteogenic differentiation potential, answering the mystery of rapid and complete regeneration of antler velvet at the cellular level [27]. However, due to immunogenicity and ethical considerations, clinical translation of xenogeneic mammalian cells is almost impossible. In addition, previous studies have demonstrated the presence of a number of bone-enhancing active factors in antler velvet [30]. Therefore, antler-derived bone replacement materials for bone defect repair have received increasing attention and have yielded promising results [[31], [32], [33]]. Li et al. demonstrated that antler decellularized matrix is more conducive to bone repair compared to cancellous bone, mainly attributed to its composition, including inorganic and organic compounds [34]. These studies mainly focused on the biological activities of antler decellularized matrix, indicating that how to maximize the retention of antler bone promoting activity while removing immunogenicity is an important research direction. Although antler is bone in nature, we found that unlike the isotropic structure of human cancellous bone, antler has typical heterogenous structure. This unique longitudinal tubular, horizontally interconnected structure of the antler is developmentally continuous and does not change much during different stages of antler development, which provides nutrient and waste metabolism pathways for rapid growth and serves as a framework to ensure that their growth is highly organized [35]. However, according to our literature review, whether the unique topology of deer antler facilitates LBCD repair remains unanswered.

Therefore, in the present study, we wanted to investigate whether the unique structure of deer antlers has an independent facilitating effect on the repair of LCBD. To remove cellular interference, we used a systematic decellularization treatment. To remove the interference of factors contributing to bone bioactivity, we applied strong oxidants and high temperature treatment. In addition, we excluded the interference of other components only by adjusting the matrix preparation Angle. In this study, we prepared two antler matrices parallel and perpendicular to the long axis of the antler (to achieve alignment or perpendicularity to the long axis of the long bone) and used antler cancellous bone matrix (with isotropic irregular structure) as a control. In in vitro experiments, the guiding effect of the structure on the cells was observed by observing the morphology of the cells and the depth of invasion. In in vivo experiments, the effects of the structures on soft tissue regeneration were observed by intramuscular implantation experiments, and the effects of the structures on bone regeneration and the shielding effect on soft tissues were judged by a rat segmental bone defect model.

## 2 Methods and methods

### 2.1 Preparation of matrix scaffolds

Velvet antlers were taken from sika deer when the antlers were cast for about 50 days. A ring drill with an inner diameter of 4 mm was used to extract cylindrical samples, each measuring 4 mm in diameter and 7 mm in height, from the base of the velvet antler. The samples were taken longitudinally or horizontally to the antler's long axis, and were labeled as L and H, respectively. The cylinder was obtained from the cancellous bone region of the distal femur of deer by the same method and labeled as R Matrix scaffolds was prepared using H_2_O_2_ method to remove immunogenicity and potential osteoinductive activity while preserving the matrix structure [36]. Briefly, cylindrical samples were degreased using a 1:1 mixture of chloroform methanol and remove cellular components with triton solution and nuclease. And then placed in a 5 % H_2_O_2_ solution at 37 °C and the solution was changed every 6h for a total of 12h. Samples were rinsed repeatedly with saline and dried at room temperature. Finally, 75 % ethanol was used to clean the organic solvent and sterilise the matrix scaffolds. Dry at 50 °C and set aside.

### 2.2 Characterisation of matrix scaffolds

**Histological analysis:** Native samples and matrix scaffolds were fixed for 24h in 4 % neutral buffered formalin solution in PBS at room temperature, decalcification with 10 % EDTA, were embedded in paraffin and were sectioned into 5 μm slices. The sections were deparaffinized, rehydrated and washed in distilled water. The slides underwent histological evaluation using hematoxylin and eosin (H&E) staining and Masson staining (Bei jing Solarbio Science & Technology Co., Ltd.) to observe the cellular components and general structure of the scaffolds. DAPI staining of native samples and matrix scaffolds was performed to determine the residual cell nuclei and thus the residual cellular by fluorescence microscopy (RVL-100-M, ECHO). Bone Morphogenetic Protein-2 (BMP-2) immunohistochemical staining were used to assess the residues of bioactive factors in matrix scaffolds. Briefly, after incubated with primary antibodies (Abcam, ab214821) at 4 °C overnight and washed by PBS, sections were incubated with horseradish peroxidase-linked secondary antibodies for 1.5 h and then visualized with 3, 3-diaminobenzidine solution.

**DNA and RNA quantification:** The total genomic DNA and RNA in the samples was extracted using a Genomic DNA Extraction Kit (TaKaRa, China) according to the manufacturer's instructions. Briefly, both the native samples (n = 3) and matrix scaffolds (n = 3) were lyophilized to achieve a constant weight, powdered with a grinder, and then were weighed. The extracted genomic DNA and RNA was quantified by measuring the absorbance in a spectrophotometer (Bio-Rad, USA), and normalized to the dry weight of the sample and expressed as ng/mg.

**Scanning electron microscopy (SEM):** The structure of matrix scaffolds was qualitatively evaluated by SEM. The matrix scaffolds were dried in freeze dryer. Subsequently, these dry samples were coated under vacuum with platinum alloy at thickness of 25 nm and were immediately flash carbon coated under vacuum. The samples were observed with a SU8600 scanning electron microscope (HITACHI, Japan).

**Micro-computed tomography (micro-CT):** The structure of the matrix scaffolds was further evaluated by micro-CT. Three samples of every type of matrix scaffold were scanned using a micro-CT scanner (Skyscan1076, Bruker, Belgium) with a layer thickness of 18 μm and were reconstructed using commercial software (Ctvol). Porosity was defined as the ratio of the volume of pores to the total volume. Pore size was defined as the diameter of the largest virtual sphere inside the porous scaffolds.

**Mechanical testing:** Uniaxial compression tests were performed to compare the mechanical properties of the different scaffolds. A universal testing machine (Sinter, Chang Chun, China) was used at a 1 mm/min loading rate, and only vertical movement was allowed. Load-displacement data were recorded during each compression test. Stress-strain curves were translated using Origin (OriginPro 2022, OriginLab, USA). The diameter of each cylinder was measured as the nominal cross-section for calculating the stresses, and the strain of the tested specimen was derived from the displacement of the loading crosshead. The Elastic modulus of the scaffolds was determined from the linear deformation region at the beginning of the stress-strain curve. The yield strength was determined by 0.2 % residual deformation. Three specimens in each group were tested (n = 3).

### 2.3 Cell isolation and culture

The study was approved by the Animal Ethics Committee of Basic Medical College, Jilin University. rBMSCs were obtained from 3-week-old Sprague-Dawley (SD) rats. In brief: after euthanasia of rats, the femur and humerus were separated under sterile conditions. The bone marrow was flushed out using a syringe filled with basal media (F12: DMEM 1:1 (Bioland, China), supplemented with 10 % fetal bovine serum (NEST Biotechnology) and 1 % penicillin–streptomycin). The cell mixture was plated in culture dishes and incubated with the same medium at 37 °C and 5 % CO_2_. The medium was changed after 48h to remove non-adherent cells and passaging was performed when a confluency of 90 % was reached. Experiments were performed at passage 2–3.

### 2.4 In vitro analysis of biotoxicity and biological activity

**Live/Dead Assay:** The matrix scaffolds were collected at day 2 and stained using a live/dead assay kit (BestBio, Shanghai, China). Briefly, the rBMSCs were seeded on the matrix scaffolds (L, H, and R) at a density of 1 × 10^4^ well^−1^ in 48-well culture plates to evaluate the biotoxicity. The medium was renewed every 48 h. The scaffolds were transferred to new 48-well culture plates at day 3. Calcein-AM and PI were used for staining the live and dead cells per the manufacturer's protocols. Then, the scaffolds were taken out from the wells and observed under fluorescence microscopy (RVL-100-M, ECHO).

**Cell Proliferation:** CCK-8 assay were performed at predetermined time points (1, 4, and 7 d) to evaluate the cell proliferation using cell count kit-8 (Bioland, China). Matrix scaffolds were ground to powder in liquid nitrogen, the extracts were obtained using a medium soak for 7 days and were decontaminated after centrifugation using a 0.22 μm filter. The rBMSCs were seeded in 48-well culture plates at a density of 5 × 10^3^ well^−1^ evaluate the proliferative activity. After 12 h of cell apposition, the substrate extraction solution was switched, with complete medium as a control. 10 % CCK-8 solution in the complete medium was switched at predetermined time points, and the cells were incubated at 37 °C for 2h. Then, 100 μl of the reaction solution was transferred into a new 96-well plate, and each group's optical density (OD) values were measured at 450 nm by a microplate reader.

**Alkaline phosphatase (ALP) staining:** Alkaline phosphatase assay was performed at day 7 to evaluate the osteogenic differentiation using BCIP/NBT ALP Color Development Kit (Beyotime, Shanghai, China). The extracts were obtained using the same method, and osteogenic medium was used as a positive control and complete medium as a negative control. Briefly, samples were washed with PBS, fixed with 4 % paraformaldehyde, and incubated with staining working solution for the same time at room temperature and protected from light according to the instructions. The results were observed under a stereomicroscope (Huizhida, China).

**Immunocytochemistry:** Immunocytochemical staining was performed to evaluate the expression of runt-related transcription factor 2 (RUNX-2). The extracts were obtained using the same method, and osteogenic medium was used as a positive control and complete medium as a negative control. The samples were fixed with 10 % paraformaldehyde at room temperature for 10 min, followed by cell permeabilization with 0.1 % Triton X-100 at room temperature for 20 min. To block nonspecific binding, cells were incubated with 10 % goat serum at room temperature for 30 min. Subsequently, primary antibodies against RUNX2 (A2851, AB clonal) diluted at 1:200 were added and incubated overnight at 4 °C. Secondary antibodies (SA00013-4, Proteintech) diluted at 1:400 were then added and incubated for 1 h at 37 °C. Subsequently, FITC-phalloidin and DAPI staining were performed. Finally, the images were observed and captured using a fluorescence microscope (RVL-100-M, ECHO)**.**

**Wound healing assay:** BMSCs were seeded in 12-well plates at a density of 1 × 10^5^ per well respectively. When cells confluence reached 100 %, a straight line through the middle of cells was drawn in each well using a sterile gun tip. The medium was replaced with an extraction with serum-free medium. Initial and healing images of the scratches were taken with an inverted microscope (Olympus) at 0 and 24h, respectively. The distance of the scratch before and after healing was measured using ImageJ (version 1.54), and the rate of scratch wound healing was calculated.

### 2.5 In vitro analyses of structure-guided cellular morphology and migration

**Structure-guided cellular morphology:** The BMSCs were seeded on the matrix scaffolds (L, H, and R) at a density of 1 × 106 well^−1^ in 6-well culture plate. Incubate for 3h to ensure that the cells adhere to the matrix scaffolds. The matrix scaffolds were then transferred to a new 24-well plate and incubated with complete medium for 3 days. The filamentous actin (F-actin) was stained with fluorescein isothiocyanate (FITC)-phalloidin (Yeasen, Shanghai, China), while nuclei were counterstained with stained with 4′,6-Diamidino-2-phenylindol (DAPI). Then, the scaffolds were taken out from the wells and observed under fluorescence microscopy (RVL-100-M, ECHO). The orientations of BMSCs were evaluated using the ImageJ plug-in FibrilTool, following a previously described method [37,38]. Orientation angle is the difference between the overall fibre orientation of the Region of Interest (ROI) and the horizontal axis. The anisotropic degrees were in the range of 0 (indicating no orientation) to 1 (indicating perfect orientation).

**Scaffold-guided cellular migration:** The cell layer was created based on previous studies [17], 2 × 10^5^ cells (cell suspension of 2000 cells μl^−1^ in complete medium) were seeded in the center of a custom-made silicone rings with an inner diameter of 7 mm within a 12-well plate. After 1 h of incubation, the silicone ring was removed and the dense layer of attached MSCs was washed with PBS to eliminate non-adherent cells. Matrix scaffolds (L, H, and R) were pre-wetted in complete medium and placed on top of the cell layer to allow cell migration into the scaffold and 800 μl of medium were carefully added. After 24 h, matrix scaffolds were then transferred to a new 24-well plate and incubated with complete medium for 3 days. It was ensured that the cell layer was on the bottom side during the whole experiment. Scaffolds were fixed 2 h post transfer in 4 % paraformaldehyde (PFA). The scaffolds were cut down the middle and stained with FITC-phalloidin. Overview images were recorded via fluorescence microscope and positions of cells relative to the scaffold surface were analyzed via digital image analysis using ImageJ software.

### 2.6 In vivo analysis

The guidelines of the Institutional Animal Care and Use Committee of China were followed. And the animal experiments were approved by the Animal Ethics Committee of Basic Medical College, Jilin University. The animals used were male Sprague–Dawley rats weighing between 350 and 450g. Fifteen rats were randomly divided into five groups (blank, L, H, R, and autologous bone) for segmental bone defect experiments. Two rats were used for heterotopic implantation experiments. After induction with 3 % isoflurane gas, rats were anaesthetised with 40 mg kg^−1^ of 2 % sodium pentobarbital by intraperitoneal injection. Anaesthesia was followed by shaving and preparation of the skin, disinfection with iodophor, and local anaesthesia with lidocaine. After implantation of the matrix scaffold, the wound was closed layer by layer with 3.0 absorbable sutures. Keep the animal warm until it wakes up and allow it to move freely inside the cages.

**Heterotopic implantation experiments:** Heterotopic implantation was used to assess the immunogenicity and osteoinductive activity of matrix scaffolds [27,39]. Briefly, the matrix scaffold was implanted into the gluteal muscle after the rats were anaesthetised. Three matrix scaffolds (L, H, and R) were implanted on each side. Rats were sacrificed 6 weeks after implantation to remove samples. The samples were stained with H&E to visualize fibrous tissue. The acquired image was divided into 8∗8 small ROI, and the overall anisotropy and orientation angle of each ROI were quantitatively analyzed using the ImageJ plug-in FibrilTool. For the matrix scaffold and its surrounding fibrous tissue, five randomly selected ROI were quantified using the same methodology. Correlation between scaffold and fibrous tissue orientation angles was analyzed by Pearson correlation analysis using prism software.

**Surgery for in situ bone defects in long bones:** Rats were immobilised in the lateral position and a lateral approach was used to bluntly separate the superficial gluteal muscle from the quadriceps muscle to expose the femur. A 27 mm long 6-hole locking titanium plate was initially fixed to the femur using two bone holders. 1.2 mm Kirschner needles were used for the initial drilling of holes. Four 1.5 mm self-tapping locking titanium screws secure the plate to the femur. A 0.3 mm thick wire saw was used to create a segmental bone defect of about 7 mm in the femur. And then the groups of matrix scaffolds (L, H, and R) were pressed and fixed along the long axis of the bone defect. Autogenous bone was the cut femur crushed under sterile conditions and implanted back in situ. Postoperative X-rays to ensure correct implant position (Fig. 1).

**Fig. 1:**
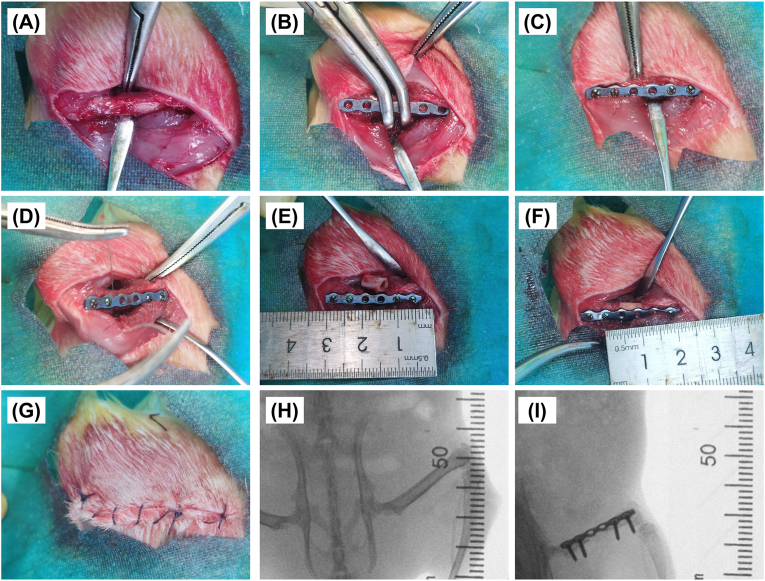
Surgical procedure. (A) Exposure of the femur. (B) Initial fixation. (C) Screw fixation. (D–E) Creation of the defect with a wire saw. (F) Implantation of a matrix scaffold. (G) Completion of sutures. (H) Preoperative x-ray. (I) Postoperative x-ray.

**Radiological evaluation:** Four weeks after surgery, the rats were sacrificed by over-anaesthesia. The femur was removed and immediately evaluated radiographically. And later, the samples were fixed with 4 % paraformaldehyde and evaluated for micro-CT. Bone volume (BV) and total volume (TV) were measured according to the gray values. The ratio of BV to TV (BV/TV) was calculated to evaluate bone ingrowth performance quantitatively [23].

**Histological evaluation:** After micro-CT evaluation, samples were decalcification with 10 % EDTA, dehydrated with a graded alcohol series and finally embedded in paraffin and were sectioned into 5 μm slices. The sections were deparaffinized, rehydrated and washed in distilled water. The slides underwent histological evaluation using hematoxylin and eosin (H&E) staining to investigate the bone ingrowth into the matrix scaffolds. The acquired images were processed with ImageJ to measure the depth of new bone ingrowth and to count the number of fibroblasts. In addition, similar to the method used for fibrous tissue analysis, we also analyzed the difference between the orientation of collagen fibers of the new bone tissue and the long axis of the femur.

### 2.7 Statistical analysis

Data were expressed as the means ± standard deviations in all experiments. One-way analysis of variance (ANOVA) was performed to compare the experimental results. GraphPad Prism 9 was utilized for all statistical analyses. And p < 0.05 was regarded as statistically significant.

## 3 Results and discussion

### 3.1 Matrix scaffolds are not immunogenic, bioactive or biotoxic

Natural velvet antler samples are filled with blood and appear red, while cancellous bone samples are filled with fat and appear white. After treatment, all samples were changed to white porous matrix scaffolds (Fig. 2A). The HE results showed a large amount of loose connective or adipose tissue around the matrix in the natural samples and also a large cellular component within the matrix. In contrast, the matrix scaffolds obtained after the series of treatments were not surrounded by other tissues, and the cells within the matrix scaffolds were also removed (Fig. 2A). DAPI staining showed that the natural samples were covered with nuclei, whereas the matrix scaffolds did not have any nuclei, further validating the results of HE staining (Fig. 2A). Moreover, DNA and RNA content in the matrix scaffolds were also quantified (Fig. 2B). After a series of treatments, the DNA content of velvet antler was reduced from 1244.31 ± 19.20 ng/mg to 19.20 ± 1.16 ng/mg and the RNA content from 1530.77 ± 14.92 ng/mg to 13.64 ± 1.40 ng/mg. The DNA content of cancellous bone was reduced from 834.92 ± 12.15 ng/mg to 15.31 ± 0.51 ng/mg and the RNA content from 751.41 ± 41.61 ng/mg to 12.02 ± 0.96 ng/mg. For decellularized matrix, when the nucleic acid content is less than 50 ng/mg, it can be assumed that most of the cellular components of the tissue have been removed and meet the implantation requirements [40]. These results above indicate that the immunogenicity of our prepared matrix scaffolds has been removed.

**Fig. 2:**
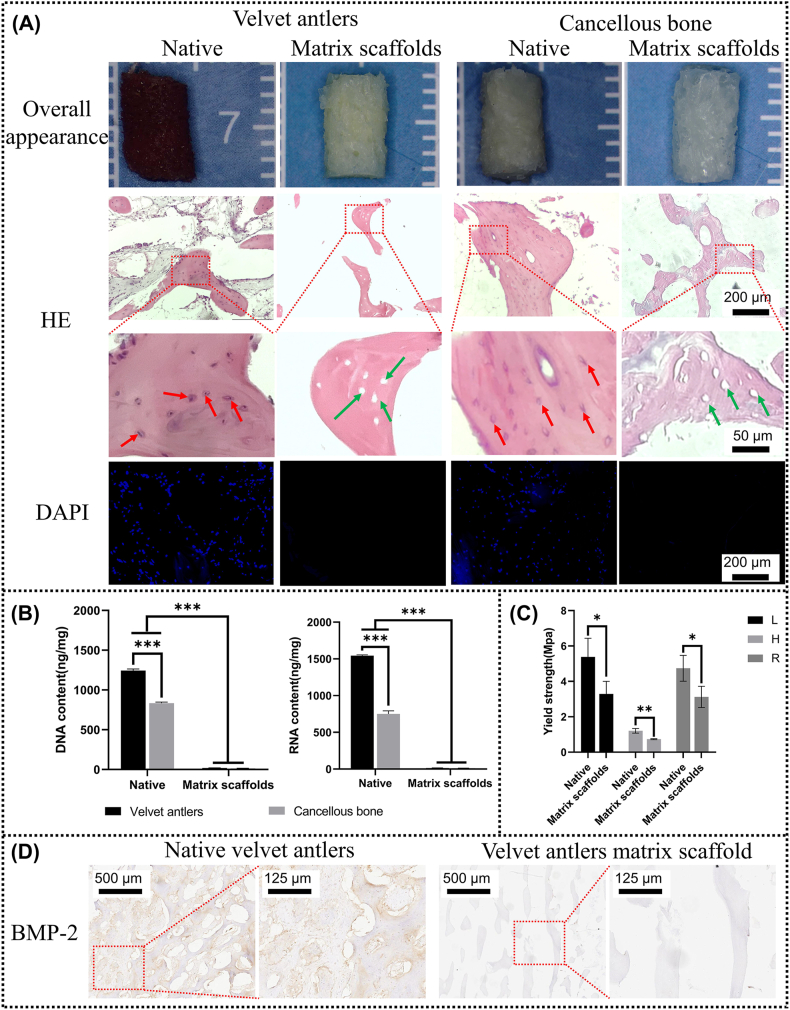
Preparation of matrix scaffolds. (A) External images, HE staining and DAPI staining of samples before and after treatment (Red arrows point to representative cells in fresh tissue, green arrows point to the decellularized cavities in the matrix scaffold, and blue fluorescent spots represent cells). (B) Results of quantitative analysis of DNA and RNA before and after sample treatment (n = 3). (C) Elastic modulus before and after sample treatment (n = 3). (D) Immunostaining for BMP-2. (For interpretation of the references to colour in this figure legend, the reader is referred to the Web version of this article.)

Masson staining and HE staining also showed that the basic structure of the samples was still existed after the series of treatments (Fig. 2A&S1). Mechanical tests were also performed on each group of samples before and after treatment (Fig. 2C). The results showed that the prepared matrix scaffolds still preserved most of the mechanical strength of the natural samples (L: 61.2 %, H: 60.8 %, and R: 63.1 %, respectively). These results suggest that the structure of the matrix scaffold is still preserved and still has the ability to maintain that structure.

In order to remove possible bone-contributing active components from natural samples to exclude interference, we utilized a variety of solvents, both polar and non-polar, repeated rinsing, and treatment with strong oxidising agents and relatively high temperatures. This was initially confirmed by immunohistochemical results against BMP-2. Natural antler tissues were strongly positive for BMP-2, whereas antler matrix scaffolds showed complete negativity for BMP-2 (Fig. 2D). Deer antler certainly does not only contain BMP-2 as an active factor, but we just use BMP-2 as a “marker” for the antler active factor library.

**Fig. 3:**
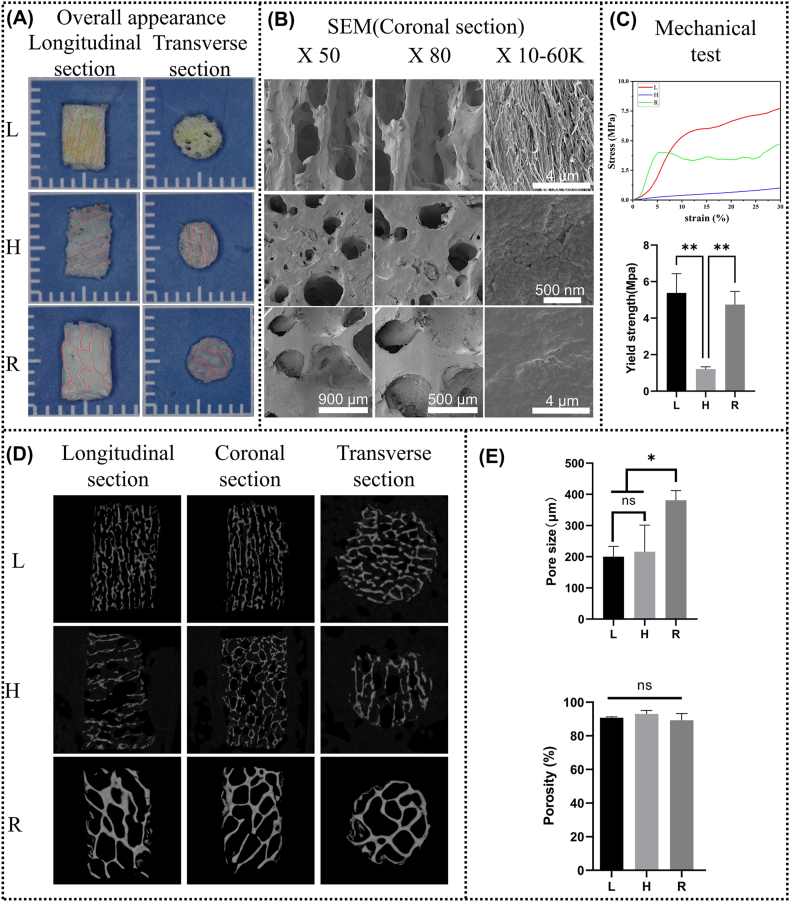
The prepared matrix scaffolds were not biotoxic or bioactive. (A) Live/Dead staining on various matrix scaffolds (Day2). (B) Proliferation of BMSCs after 1, 4 and 7 days of culture (n = 3). (C) The ALP staining results after 7 days of culture. (D) Immunofluorescence co-staining results for F-actin (green), DAPI (blue) and RUNX-2 (red) after 7 days of culture. (ns = no significance; C = control i.e. complete medium; C- = negative control i.e. complete medium; C+ = positive control i.e. osteoinduction medium). (For interpretation of the references to colour in this figure legend, the reader is referred to the Web version of this article.)

The matrix scaffolds were further assessed by a series of in vitro experiments to determine whether they were biotoxic and whether the biological activity was completely removed. Fig. 3A showed the results of live and dead fluorescence staining of three groups of matrix scaffolds, where live cells were stained with green fluorescence and dead cells with red fluorescence. All three groups of matrix scaffolds were dominated by living cells, with almost no dead cells, showing All three groups of matrix scaffolds were dominated by living cells, with almost no dead cells, indicating that the prepared matrix scaffolds were not biotoxicity. The CCK-8 assay was used to detect the effect of biologically active or toxic components on cell proliferation. As shown in Fig. 3B, in terms of cell proliferation trends, all groups showed changes in cell number growth over time, indicating the normal proliferative capacity of the cells. In addition, at each time point, there was no statistically significant difference in the number of cells in each group compared to the control group, suggesting that we removed the bioactive substances within the antler or bone and that no toxic substances remained.

ALP staining and RUNX-2 immunohistochemical staining were performed to determine whether we had removed the osteoinductive activity of the matrix scaffolds. ALP promotes phosphate release and bone matrix maturation by hydrolysing organophosphate compounds, playing a key role in bone formation and maintenance, and is often used as a marker of osteogenic differentiation [41]. As shown in Fig. 3C, the osteogenic induction medium group (C+) showed strong positivity for ALP, whereas there was no significant difference between BMSCs cultured in matrix scaffold extract in all groups and complete medium (C-), which were all negative. Runx-2, a transcription factor that plays an important role in the early differentiation stages of osteoblasts, activates the expression of a range of genes associated with osteogenesis, thereby driving the differentiation of mesenchymal stem cells into the osteoblast lineage [42]. Fig. 3D shows similar results to the alp staining, i.e. the osteoinductive activity of the matrix scaffolds in each group has been removed.

It has been widely documented that antler velvet contains many active substances that contribute to bone or promote cell proliferation [43,44]. It is worth noting that having activity is undoubtedly a favorable factor for osteogenesis, and our aim in removing matrix scaffold activity was to preclude their interference with subsequent studies.

### 3.2 Significant differences in orientation of matrix scaffolds

**Fig. 4:**
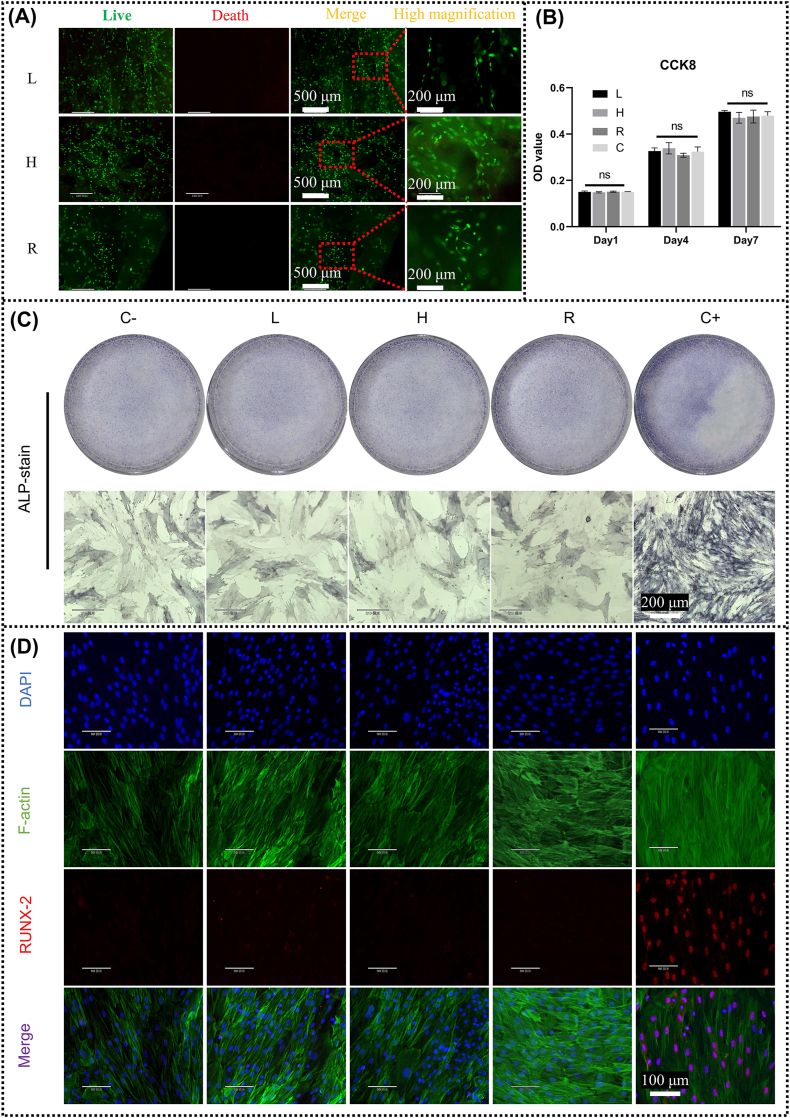
The Structural features of matrix scaffolds. (A) Overall appearance of matrix scaffolds. (B) The SEM images of the coronal section of matrix scaffolds. (C) Representative stress-strain curves of matrix scaffolds and the yield strength of matrix scaffolds. (D) The micro-CT images of matrix scaffolds. (E) Quantitative analysis of pore size and porosity of matrix scaffolds in each group. (∗P < 0.05, ∗∗P < 0.01).

The three groups of L, H, and R scaffolds have significant differences in orientation. As shown in Fig. 4A, the longitudinal section of the L matrix scaffold (parallel to the antler's long axis) showed longitudinal channels, while the transverse section exhibited circular pores. In contrast, the longitudinal section of the H matrix scaffold (perpendicular to the antler's long axis) showed transverse channels, while the transverse section also exhibited channels. Unlike antler matrix scaffolds, cancellous bone matrix scaffolds were irregularly structured in both longitudinal and transverse sections. Fig. 4B shows SEM images of the coronal section of each group of matrix scaffolds. At high magnification, L-matrix scaffold showed highly orderly arranged collagen fibers, and H-matrix scaffold showed the cross-sections of these collagen fibers, while no directional arrangement of collagen fibers was observed in the R-matrix scaffold. And at low magnification, the channels and pores of L and H could be observed clearly. Notably in the coronal section of group L, we could see that the channels are not isolated and closed, but interconnected with the surrounding channels through small pores. Therefore, we tentatively concluded that antlers have a structure of “longitudinal tubular transverse interconnection”.

Micro-CT allows the structure of the matrix scaffolds to be viewed at multiple levels and from multiple angles. As shown in Fig. 4C, the L matrix scaffold was approximately circular in transverse section and tubular in both the coronal and longitudinal sections. In contrast, the H matrix scaffold exhibited transverse tubular structure in the longitudinal section and was approximately circular in the coronal section. R matrix scaffolds, on the other hand, were randomly irregular in all three cross-sections. In addition, both L and H showed that the tubular structures of antler matrix scaffolds were interconnected rather than closed. The site of extraction is identical between L and H. The only difference between the two is the direction of extraction, which also results in completely different mechanical properties. As shown in Fig. 4D, the yield strength of L matrix scaffold reached 4.5 times that of the H matrix scaffold, which fully justifies the difference in structural orientation between the two [45].

Fig. 4E showed the porosity and pore size of the three sets of matrix scaffolds, which are important structural factors affecting bone regeneration [23]. There was no statistical difference between the pore size of the L matrix scaffolds (200 ± 32.96 μm) and the pore size of the H matrix scaffolds (215.8 ± 85.79 μm). The pore size of the R matrix scaffold (380.9 ± 30.78 μm) was significantly larger than that of the L and H. There was no significant difference in porosity between the three groups (L:90.77 ± 0.52 %, H: 93.05 ± 2.11 %, and R: 89.30 ± 3.985 %).

### 3.3 The structure of the matrix scaffolds guided the orientation and migration of cells

**Fig. 5:**
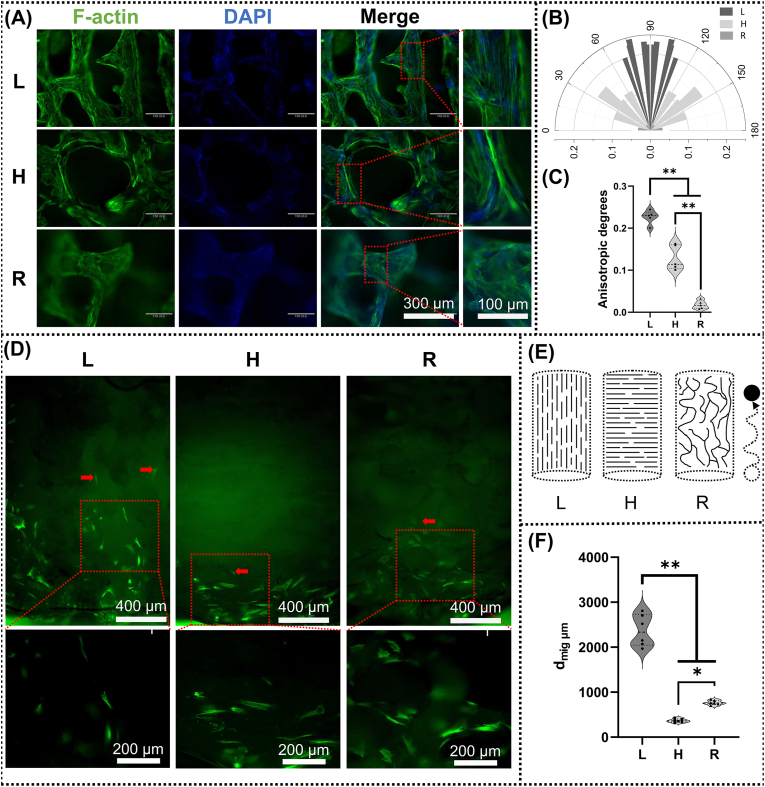
The structure-guided cellular morphology and migration. (A) Fluorescent images of BMSCs morphologies on matrix scaffolds after being cultured for 3 days. The Cytoskeleton was shown as green fluorescence, and the nuclei were shown as blue dots. (B) Semipolar coordinate plots of BMSCs cytoskeleton orientation on each group of matrix scaffolds. Polar angle is the angle of the cytoskeleton to the horizontal axis (range from 0° to 180°). The radius is anisotropic degrees (range from 0 to 1, 0 represents complete isotropy and 1 represents complete anisotropy). (C) The anisotropic degrees of BMSCs on each group of matrix scaffolds. (n = 5) (D) longitudinal section staining for F-actin after BMSCs seeding on the base of the matrix scaffold cylinder 3 days. The bright green fluorescence represents BMSCs migrating from the bottom of the cylinder into the matrix scaffolds. Red arrows point to BMSCs with the furthest migration distances. (E) The schematic diagram of cell migration on each group of matrix scaffolds, circles represent BMSCs, arrows represent the direction of migration. (F) Cell migration distances (d_mig_) on each group of matrix scaffolds. (n = 6) (∗P < 0.05, ∗∗P < 0.01). (For interpretation of the references to colour in this figure legend, the reader is referred to the Web version of this article.)

We firstly investigated the effect of the structure of the matrix scaffold on cell morphology and cell migration. We seeded BMSC onto each group of matrix scaffolds and fluorescently stained the cytoskeleton. As shown in Fig. 5A, the arrangement of cells on the matrix scaffolds of group L was highly ordered, whereas the orientation of the cytoskeleton on the matrix scaffolds of groups H and R was relatively disorganized. Fig. 5B showed the results of semi-quantitative analyses of cytoskeleton orientation. The orientation of the cells on the matrix scaffolds in group L was highly ordered, mostly at about 90°. In contrast, the orientation of the cells on H and R was relatively disordered, with a wider range of distribution, from 0 to 180°. Fig. 5C showed the results of semi-quantitative analyses of cytoskeleton anisotropy. Anisotropic degrees were range from 0 to 1, 0 represents complete isotropy and 1 represents complete anisotropy. Cytoskeletal anisotropy on the scaffolds of the L group was significantly higher than on the H and R.

As shown in Fig. 5F, we also spread the BMSCs on the bottom of the matrix scaffolds to assess the migration distances of cells within different matrix scaffolds. As shown in Fig. 5D, cells migrated the deepest distance within the matrix scaffolds of group L. Fig. 5F showed the results of the quantitative statistics of migration distances(d_mig_), suggesting that the migration distances of the cells within the L group were significantly higher than those within the H and R groups. However, scratch experiments showed that there was no significant difference in the migration rates of cells treated with different matrix scaffold extracts (Fig. S2). These results indicated that the cause of the different depths of cell migration within the matrix scaffold was not due to the active components, but rather due to structural differences. In particular, groups L and H had identical compositions, which differed only by orientation.

### 3.4 The structure of the matrix scaffold guided tissue growth

**Fig. 6:**
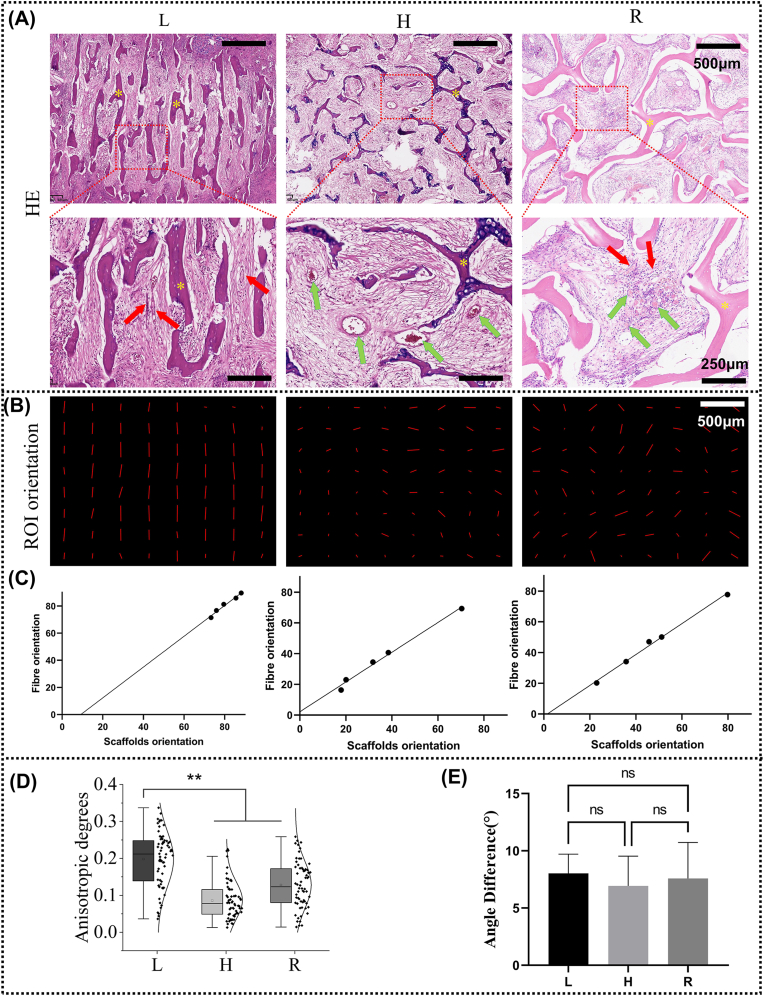
The results of heterotopic implantation experiments. (A) HE staining of matrix scaffolds 6 weeks after implantation into muscle. Yellow asterisks are matrix scaffolds. Longitudinal sections of vessels are indicated by red arrows. The cross section of the vessel is indicated by the green arrow. (B) Orientation images of ROI for L, H, and R at 6 weeks after implantation. The length of the line represents the anisotropy of the ROI and the direction represents the ROI orientation. (C) Correlation analysis between fibre orientation and scaffolds orientation (n = 5). (D) Distribution of the anisotropy of ROIs for each group (n = 64). (E) Angle difference between fibre orientation and scaffolds orientation (n = 5). (ns = no significance). (For interpretation of the references to colour in this figure legend, the reader is referred to the Web version of this article.)

The heterotopic osteogenesis assay is the gold standard for testing whether an implant has osteoinductive activity. If the implant has osteoinductive activity, newborn osteoblasts or chondrocytes are usually visible after 6 weeks of intramuscular implantation [46,47]. As shown in Fig. 6A, after 6 weeks of intramuscular implantation, the matrix scaffolds were filled with fibrous tissue in all groups and no new osteoblasts or chondrocytes were seen, further corroborating the fact that we had completely removed the bioactivity of antler or deer bone. In addition, we can also see that there is also no inflammatory response (plasma cells or monocytes) within the groups of scaffolds, indicating that we have also removed their immunogenicity.

Interestingly, we observed that the growth of fibrous tissue and blood vessels within the matrix scaffold was significantly guided by the scaffold's structure. As shown in Fig. 6A, in the L matrix scaffold, we observed the longitudinal section of blood vessels (indicated by red arrows), while in the H group scaffold, we observed the cross-section of the scaffold (indicated by green arrows). In the R scaffold, both the cross-section and longitudinal section of blood vessels were visible. These results suggests that the orientation of blood vessels aligns with the direction of the matrix scaffolds.

Furthermore, we observed that the alignment of fibrous tissue also closely reflected the orientation of the scaffold structure. As shown in Fig. 6B, HE-stained sections were divided into 64 regions of interest (ROIs), and the anisotropy and orientation within each ROI were analyzed. The results demonstrated that fibrous tissue exhibited high isotropy in the longitudinal sections of the deer antler matrix scaffold, whereas anisotropy was observed in the transverse sections of the deer antler matrix scaffold and throughout the deer bone matrix scaffold (Fig. 6B and D). Additionally, we randomly selected five ROIs to analyze the orientation of the matrix scaffold and adjacent fibrous tissue. The results revealed a linear correlation between fibrous tissue orientation and scaffold orientation across all three groups (Fig. 6C), with no significant difference in the angle difference between them (Fig. 6E). These findings collectively indicate that tissue growth, including blood vessels and fibrous tissue, within the matrix scaffold is significantly influenced by its structural architecture.

### 3.5 Antler matrix scaffolds accelerate regeneration of large segmental bone defects in long bones

We constructed a rat femur standardized large segmental bone defect model to assess the effect of the structure of antler matrix scaffolds in bone regeneration. After four weeks, it can be seen from the gross external image that the newborn bone callus integrated more tightly with the matrix scaffold in the L group. And after removing the plate, external force applied by hand could not produce micro-movement between the host bone and the matrix scaffold. In contrast, the medullary cavity in the blank group had been closed, implying bone discontinuity, which would inevitably lead to the separation of the two stumps once the steel plate was removed (Fig. S3A). Micro-CT provided a good window to observe the nascent bone at all levels inside the matrix scaffold. As shown in Fig. S3C, the newborn bone within the matrix scaffold in group L grew in deeper than the other two groups.

**Fig. 7:**
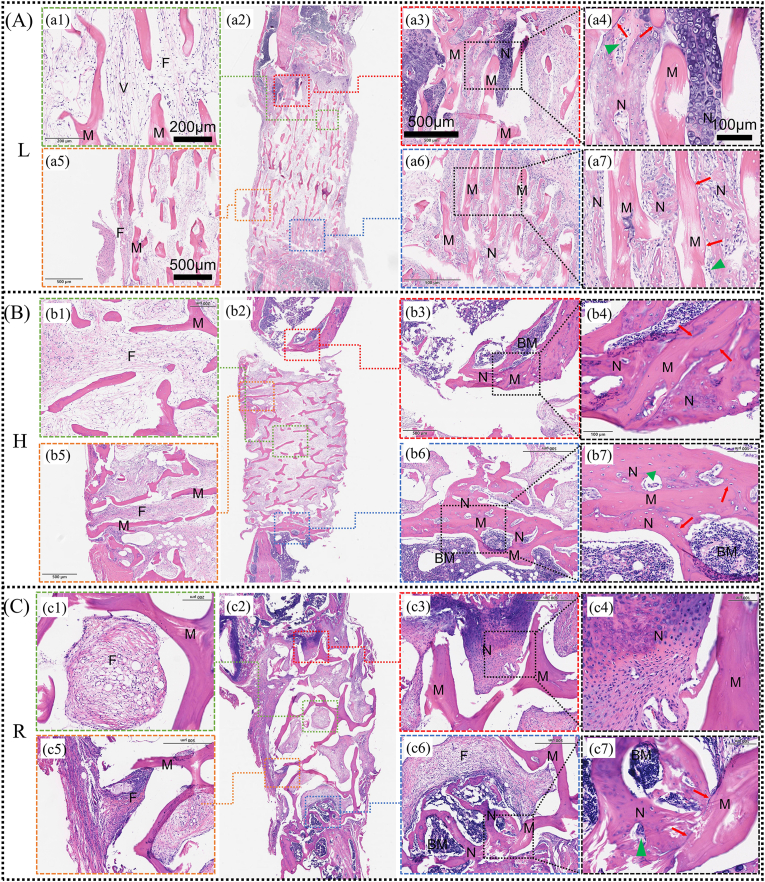
Histological results of in situ implantation experiments. (A–C) HE-stained images of three groups of matrix scaffolds implanted in the mid-femur of rats 4 weeks after implantation and their local magnification images. (a1-c1) Fibrous tissue inside three groups of matrix scaffolds (bar = 200 μm). (a5-c5) Fibrous tissue around three groups of matrix scaffolds (bar = 500 μm). (a3-c3) Bone tissue regeneration at the proximal end of the matrix scaffold (bar = 500 μm). (a6-c6) Bone tissue regeneration at the distal end of the matrix scaffold (bar = 500 μm). (a4-c4 and a7-c7) Local magnification images of the matrix scaffold at the junction with host bone (bar = 100 μm). M = Matrix scaffolds; F=Fibrous tissue; V=Vasculature; N=New bone regeneration; BM=Bone marrow. Green triangles point to osteoclasts, red arrows point to mineralization line. (For interpretation of the references to colour in this figure legend, the reader is referred to the Web version of this article.)

Histologic staining is the gold standard for diagnosing newborn bone situations. As shown in Fig. 7, there was no significant inflammatory cell infiltration or cell necrosis in or around the scaffolds in any of the groups, proving once again that we removed the immunogenicity of antler as well as deer bone and that the implants were nontoxic. At the same time, the osteoclasts were observed in the matrix scaffolds of each group (Green triangles in Fig. 7), and some of them were located between the matrix scaffold and the new bone tissue, indicating that the matrix scaffold could be degraded by the body. As shown in Fig. 7a5, the L-matrix scaffold resisted the invasion of most surrounding fibrous tissues, with relatively loose fibrous organization within its structure (Fig. 7a1). In contrast, fibrous tissues surrounding the H-matrix scaffold infiltrated smoothly through transverse channels into the bone defect (Fig. 7b5), exhibiting denser fibrous organization within the defect (Fig. 7b1). The soft tissue invasion pattern in R-matrix scaffolds was intermediate between these two groups. These results paralleled ectopic implantation experiments, demonstrating that fibrous tissues preferentially aligned with the scaffold's structural orientation. In the L-group, longitudinal channel alignment with the bone stump effectively blocked fibrous tissue penetration.

The bone regeneration pattern of all three groups of scaffolds grew from the ends to the middle, which indicated that the matrix scaffolds did not have osteoinductive activity, and the pathway of their repair of bone defects was osteoconduction (Fig. 7a2b2c2). In addition, the varying degrees of chondrocytes in the high magnification view indicated that the mode of osteogenesis was endochondral osteogenesis (Fig. 7a4a7c4). Notably, significant differences in bone ingrowth depth were observed among the three matrix scaffolds. Bone within the L matrix scaffold grew in the deepest (Fig. 7a3a6), neoplastic bone within the H matrix scaffold was essentially blocked by the matrix scaffold (Fig. b3b6), and bone within the R scaffold grew in at a depth in between. Intriguingly, mature neobone with marrow cavities and minimal chondrocytes was observed at both ends of H-matrix scaffolds. In contrast, L-matrix scaffolds contained immature new bone with abundant chondrocytes, suggesting ongoing regenerative potential. R-matrix scaffolds displayed chondrocyte presence constrained by transverse trabeculae, limiting further invasion.

**Fig. 8:**
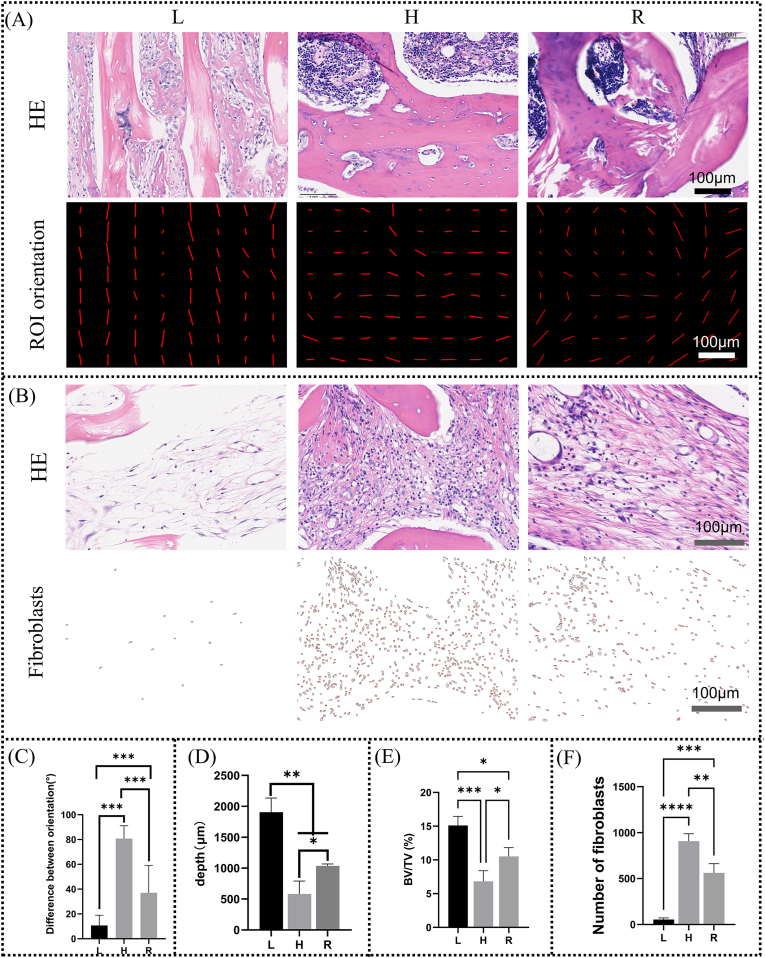
Quantitative analysis of new bone formation and soft tissue invasion in the matrix scaffold. (A) Representative histological images of the new bone within the matrix scaffold and orientation images of ROI for L, H, and R at 4 weeks after implantation at the defect site. The length of the line represents the anisotropy of the ROI and the direction represents the ROI orientation. (B) Representative histological images of soft tissue within the matrix scaffold and fibroblasts images of ROI for L, H, and R at 4 weeks after implantation at the defect site. Each small brown-red dot represents a fibroblast. (C) The difference between ROI orientation and long-axis orientation of the femur. (D)Depth of bone growth into different matrix scaffolds. (E) Analysis of bone volume fraction at the defect site based on Micro-CT. (F) Counting analysis of the number of fibroblasts within the ROI region. (For interpretation of the references to colour in this figure legend, the reader is referred to the Web version of this article.)

Quantitative analyses were performed on the depth, volume and orientation of new bone formation, and the degree of soft tissue invasion. As shown in Fig. 8A and C, the orientation of the new bone within the L matrix scaffold was essentially parallel to the long axis of the femur, whereas the orientation within the H matrix scaffold was essentially perpendicular to the long axis of the femur, and the orientation within the R group matrix scaffold was in between. Bone ingrowth depth was significantly greater in L-scaffolds compared to other groups, with H-scaffolds showing minimal penetration (Fig. 8D). Micro-CT quantification confirmed superior bone volume in L-scaffolds and minimal volume in H-scaffolds (Fig. 8E). It is worth noting that this value does not reflect the absolute value of new bone tissue within the scaffold, as the matrix scaffold has inherent radiopacity. However, considering that there is no difference in the radiopacity of the three scaffolds themselves (Fig. 4E), we can still infer from this result that the L matrix scaffold has the highest volume of new bone tissue. Fig. 8B showed representative HE sections of the soft tissue within the matrix scaffold and the distribution of fibroblasts separately for each group. Cell counting showed that the number of fibroblasts in the L matrix scaffold was the least, while the H group was the most (Fig. 8F).

## 4 Discussion

For deer antler matrix scaffolds, structure is an independent factor in accelerating LBCD repair. Antlers are the only bony organ in mammals that can be completely regenerated periodically, with a growth rate of up to 2 cm per day [28]. In contrast, human bone grows at a rate of only 1 mm per day, and exceeding this rate of distraction may lead to bone nonunion [29]. Despite the fact that both antler and long bones are endochondral osteogenic processes, the difference in the rate of osteogenesis between the two is striking. In previous studies, our team identified an antler stem cell in antler velvet, which has an extremely strong proliferative capacity and osteogenic differentiation potential, answering the mystery of rapid and complete regeneration of antler velvet at the cellular level [27]. However, due to immunogenicity and ethical considerations, clinical translation of xenogeneic mammalian cells is almost impossible. With these issues in mind, decellularized matrices are increasingly of interest to researchers because they often preserve some of the bioactive components of natural tissues. But it is also for this reason that it is difficult to tell whether structure is an independent factor in achieving bone repair. As shown in 3.1 and 3.4, interference with biological activity has been shown to be completely excluded. It is worth noting that mechanical stimulation is also an important factor affecting bone regeneration, especially the orientation of the matrix scaffold causes a 4.5-fold difference in mechanical strength (L VS H). Therefore, we cannot completely rule out mechanical factors in the large differences in the repair effects of L and H in the in vivo experiments. However, in vitro experiments, the three groups of scaffolds are not stimulated by mechanical stimulation, and there are also huge differences in biological effects. And in vivo experiments, the mechanical strength of L and R was basically the same, but the repair effect was statistically different. Taking these results into account, we believe that the unique structure of the longitudinal canal transverse connection of deer antlers independently promotes the repair of large bone defects. This result has important implications not only for the development of antler-associated bone substitutes but also for the design of additively manufactured bone implants.

Many studies have shown that structure is an important factor affecting bone tissue regeneration [21,23,24]. Generally speaking, the structure mainly includes the pore size, porosity and the pore shape. Pore size may affect bone formation by influencing cell adhesion efficiency and fluid permeation. In general, small pores are more favorable for initial cell adhesion. However, smaller pore sizes are not necessarily better. Research by Hulbert et al. [48] shows that bone tissue has difficulty growing into pores smaller than tens of micrometers. Meanwhile, large pores are more favorable for nutrient penetration and waste removal. Many studies have shown that pores larger than 800 μm are not conducive to bone tissue invasion and are related to cell adhesion efficiency [23,49,50]. The optimal pore size is not consistent among different studies due to the differences in experimental animals, bone defect models, scaffold materials and pore shapes, but generally it is in the range of hundreds of microns. In our previous research, we determined that the optimal pore size for bone tissue engineering scaffolds used in cancellous bone repair is around 500 μm. Research by Keigo et al. [51] showed that pores smaller than 550 μm were more conducive to the restoration of cortical bone orientation. For this study, the pore size of the L and H matrix scaffolds was exactly the same, about 200 μm, and the pore size of the R matrix scaffold was about 400 μm, both in the range of hundreds of microns. However, L and H had the best and worst repair effects, respectively, while R was in the middle, indicating that pore size was not the core feature of antler structure. Porosity is a core parameter to evaluate the permeability of scaffolds, but there was no statistical difference in porosity among the three groups (Fig. 4E), which could not explain the different repair effects, so porosity was also not a dominant factor in this study. The pore shape then affects the curvature of the material, and in general, high curvature is favorable for bone regeneration, correlating with mechanical force signaling pathways. The trabecular structure and the triperiodic minimal surface have been widely shown to be more conducive to cancellous bone repair than the cuboidal structure [23,52]. However, L and H have exactly the same pore shape, and the only difference between the two is the different orientation of the pore. Therefore, we hypothesize that the cause of this discrepancy may be a structural parameter that has received little attention, i.e., pore orientation. Fig. 4 fully revealed the difference in orientation between the three sets of matrix scaffolds. We therefore speculate that highly anisotropic longitudinal tubular transverse connectivity is a central feature of antler matrix scaffold structure.

Tissue growth is guided by the structure of the longitudinal tubular transverse connection. In vivo, cells are guided by the complex topological structure of the extracellular matrix (ECM). For example, the distribution and migration direction of breast cancer cells are significantly affected by the diameter of collagen fibers [53]. Surface topography regulates neural stem cell migration and cell morphology [54]. The highly orderly and rapid growth of deer antler is also guided by its highly anisotropic extracellular matrix [35]. In this study, we retained the unique highly anisotropic topological structure of the extracellular matrix of antlers, with scales ranging from nanometers to micrometers (Fig. 4). Interestingly, even in vitro, the growth of cells is still guided by the structure of matrix scaffold. As shown in Fig. 5, cells are not only arranged more orderly on the L-matrix scaffold, but also invade more deeply. At the tissue level, the orientation of the newly formed fibrous tissue and blood vessels are also highly aligned with the matrix scaffold (Fig. 6). In the in-situ implantation experiment, the newly formed bone tissue was also highly aligned with the L-matrix scaffold and grew deeper (Fig. 8). This phenomenon might be caused by the contact guidance mechanism, which refers to the terrain cues regulating the adhesion, morphology and migration of cells [55,56]. Cells connect the cytoskeleton to the extracellular matrix through structures such as adhesion plaques, while the asymmetric terrain pattern (anisotropy) leads to local asymmetric stress, eventually resulting in the transformation of cell morphology [57]. As shown in Fig. 4B, the L matrix scaffold has an anisotropic structure ranging from nanometer to micrometer scale. As shown in Fig. 5B, this anisotropy further leads to a directional arrangement of cellular stress fibers. The migration of cells depends on the thrust generated by the directional polymerization of actin and the directional movement of focal adhesion [58]. Thus, it is the highly anisotropic structure of the L matrix scaffold that changes the local stress pattern of the cells, thereby changing the orientation of the stress fibers within the cells, which in turn accelerates cell migration. These reviews summarize well how biomaterials regulate cell migration in the absence of chemokines [55,58,59]. The alignment at the tissue level should be further triggered by the initial cell alignment. Previous studies have shown that tissue form is not only a consequence but also an active regulator of tissue growth [60,61]. The tubular structure of the deer antler matrix scaffold provides restrictive conditions for the nascent tissues, which is more conducive to the height alignment of the nascent tissues.

Highly anisotropic longitudinal canal transverse connection structure facilitates shielding of fibrous tissue invasion into the bone defect site. In the case of large bone defects in long bones, only the upper and lower ends of the defect are made of bone tissue, while the surrounding area is made of fibrous tissue. Generally speaking, fibrous tissue regenerates faster than bone, and when fibrous tissue occupies the center of the defect before bone does, the new bone at both ends often fails to make contact again, and bone nonunion occurs. Based on this consideration, barrier membrane technology has received great attention in the field of dentistry, which is used to preserve osteogenic space and prevent unwanted cells from migrating from the gingival epithelium and fibrous tissue [[62], [63], [64]]. In the repair of LBCD, the induced membrane technique is more familiar to orthopedic doctors. The Induced Membrane, also known as the "Masquelet membrane", is a vascular-rich tissue membrane formed by the foreign body reaction in the body after placing bone cement spacers in the bone defect area [65]. Similar to barrier membranes, the success of the induction membrane technique is largely attributed to the preservation of the space in the bone defect area (for bone regeneration) and the shielding effect on the surrounding soft tissues.

As mentioned earlier, the deer antler matrix scaffold has a highly anisotropic longitudinal tube transverse connection structure, and various tissues, including soft tissues, are guided by it. This difference in the growth orientation of soft tissues also plays a role in shielding soft tissues from invading bone defects. As shown in Fig. 7a–c5, most of the fibrous tissue outside the L matrix scaffold was shielded outside the bone defect, the fibrous tissue outside the H matrix scaffold grew along the matrix scaffold into the bone defect, while the fibrous tissue outside the R matrix scaffold was partially shielded. Correspondingly, as shown in Fig. 7a–c1, the fibrous tissue in the L matrix scaffold is extremely loose, while the fibrous tissue in the H and R matrix scaffords is relatively dense. In addition, unlike membrane technology, the tubular structure of the deer antler matrix scaffold is more conducive to the alignment and climbing growth of new bone tissue. In the field of peripheral nerve injury repair, neural guide conduits (NGCs) are increasingly regarded as potential alternatives to autologous nerve grafts because nerves grow more slowly than bone tissue and also need to reserve growth space for them [66]. For the design of NGC, the Multi-channel design is more popular than the hollow design because Microchannels are considered to facilitate the directional alignment of neurons [66]. The antler matrix scaffolds is a naturally multi-channel design. In the past, the repair of LBCD might have focused more on the recovery of new bone mass. However, an increasing number of studies have recognized that restoring the orientation of long bones is also very important, because long bones themselves are highly anisotropic tissues, which are closely related to their strength [51]. As shown in Fig. 8, when the orientation of the deer antler matrix scaffold is aligned with the long axis of the host bone, both the amount and orientation of new bone formation inside it are significantly enhanced.

In conclusion, the antler matrix scaffold is a highly anisotropic longitudinal canal transverse connection scaffold designed by nature across scales. Many studies have achieved highly aligned textures on material surfaces at the nanoscale through lithography technology and further regulated the morphology and migration of cells [57,67]. Recently, the controlled directional freezing technology has achieved collagen scaffolds with directional pore structures ranging from tens of micrometers to about 200 μm, and has realized ordered new bone formation in both in vivo and in vitro experiments [17,68]. Additive manufacturing technology can produce directional channels of hundreds of micrometers in size and has achieved encouraging results in the repair of long bone defects [51,69]. The research of Christopher et al. [70] shows that directional cues ranging from hundreds of nanometers to hundreds of micrometers can guide the directional arrangement of cells and the resulting tissues. More importantly, when the orientations of the two are the same, they can synergistically enhance this effect, while when they intersect, competition will arise. To achieve this cross-scale alignment, Chou et al. developed a novel roll-up method, embedding the 2-D microscale structure into the 3-D structure [71]. Antler matrix scaffolds are a kind of scaffold with cross-scale and highly arranged scaffolds bestowed upon us by nature. Due to their functions of blocking soft tissue invasion and accelerating the alignment of new bones, they have broad application prospects in LBCD repair. It is worth noting that in this work, in order to eliminate the interference of biological activity and immunogenicity, we carried out thorough decellularization and oxidative inactivation treatment. In future work, we will consider how to remove immunogenicity while retaining more biological activity to accelerate the repair of LBCD.

## 5 Conclusion

Using systematic physical, chemical and biological methods, the scaffolds were successfully prepared by removing the antler cells and bioactive components. In vivo and in vitro experiments showed that they had no immunogenicity, bioactivity or biotoxicity. The structure of the antler matrix scaffold was well preserved, with longitudinal tubule-transverse connection and cross-scale anisotropy. This structure is an independent factor in accelerating LBCD repair. The effect is mainly attributed to two aspects, one is the shielding effect on the invasion of fibrous tissue, the other is to guide the orderly regeneration of new bone tissue through osteoconduction.

## CRediT authorship contribution statement

**Chenyu Wang:** Writing – review & editing, Writing – original draft, Data curation, Conceptualization. **Wenbo Yang:** Writing – review & editing, Writing – original draft, Visualization, Data curation, Conceptualization. **Lanfeng Song:** Visualization, Validation, Data curation. **Lanqing Cao:** Visualization, Data curation. **Guokun Zhang:** Resources. **Xiaofan Gao:** Writing – review & editing, Visualization. **Xiujie Zhu:** Investigation. **Shipu Jia:** Formal analysis, Data curation. **Xiang Yue:** Formal analysis. **Chunyi Li:** Resources. **Jincheng Wang:** Writing – review & editing, Resources, Funding acquisition. **Xin Zhao:** Writing – review & editing, Supervision, Funding acquisition. **Haotian Bai:** Writing – review & editing, Resources, Funding acquisition, Conceptualization.

## Statement of significance

The repair of long-bone critical defect (LBCD) remains a formidable clinical challenge due to the fibrotic microenvironment at defect sites. While autografts are current gold standards, their limited availability and complications necessitate alternative biomaterial solutions. This study pioneers a novel approach by leveraging the unique structural properties of deer antler - the only completely regenerable osseous organ in mammals, exhibiting extraordinary growth rates (2.7 cm/day vs. 1 mm/day in human long bones). Unlike previous studies focusing on bioactive components of decellularized antler matrix, we demonstrate for the first time that the structure of antler matrix alone - independent of cellular or biochemical factors - significantly enhances LBCD repair. Mechanistically, this scaffold achieves dual functionality: 1) Physically shielding soft tissue invasion through its dense surface layer, and 2) Directionally guiding neo-bone formation via its oriented channel structure. These findings fundamentally advance our understanding of mammalian bone regeneration paradigms and establish a new design principle for bone graft substitutes. The structural blueprint derived from antler regeneration offers transformative potential for developing next-generation osteoconductive implants and optimizing decellularized matrix processing protocols. This work bridges evolutionary biology with clinical orthopedics, providing an innovative roadmap to address critical-sized bone defects.

## Funding

This work was supported by the 10.13039/501100001809National Natural Science Foundation of China (grant Nos. 824B200222, U23A20523, 82202698, and 82472460); 10.13039/100007847Natural Science Foundation of Jilin Province (Grant Nos. YDZJ202201ZYTS086); Proof of concept of medical program in Jilin University (24GNYZ36, 24GNYZ37); the Jilin Province Development and Reform Commission, P.R.C (2022C044-2); the Department of Science and Technology of Jilin Province, P.R.C (20220204117YY, 20220402067 GH)

## Declaration of competing interest

The authors declare that they have no known competing financial interests or personal relationships that could have appeared to influence the work reported in this paper.

## Figures and Tables

**figs1:**
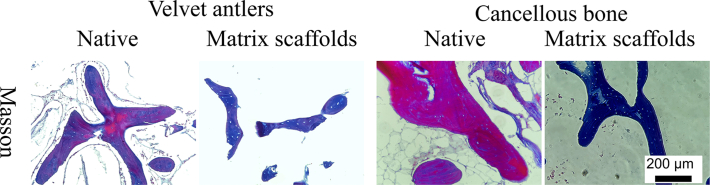


**figs2:**
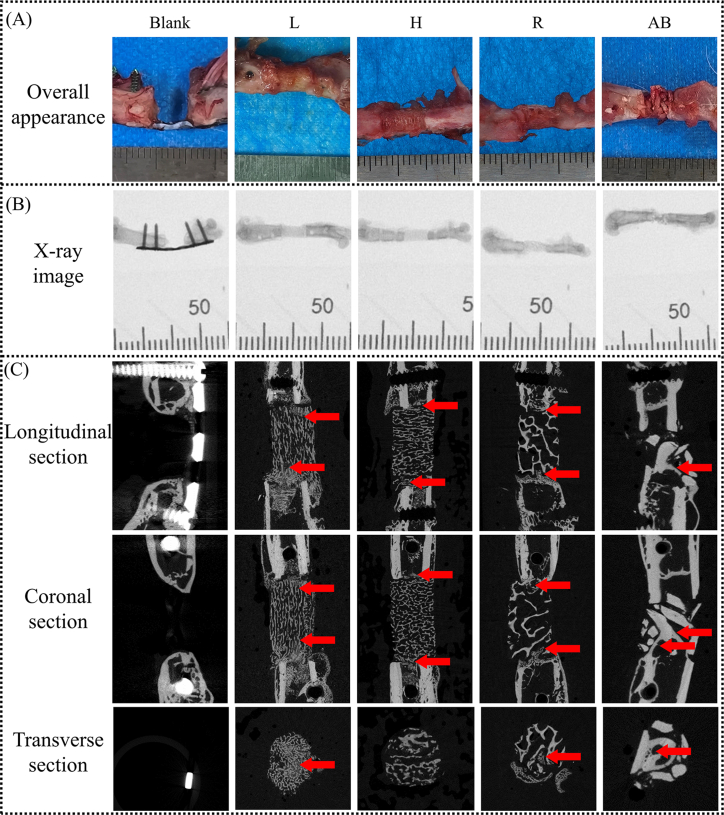


## Data Availability

Data will be made available on request.
