# Supplementary material for: An ingenious design from nature to accelerate the repair of long-bone critical defects: the longitudinal tubular transverse interconnection structure of deer antlers
Source: Mater Today Bio. 2025 Jul 15;34:102090. doi: 10.1016/j.mtbio.2025.102090 (PMC12305181; doi:10.1016/j.mtbio.2025.102090)
Supplement: Multimedia component 1 [file mmc1.docx]

Supplementary documents


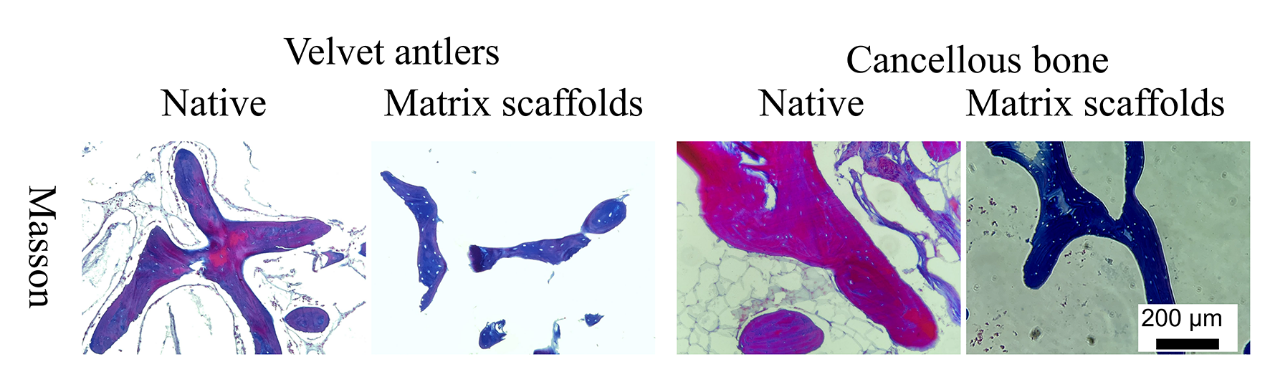


Fig. S1 The Masson stain of three groups of matrix scaffolds (n=3).


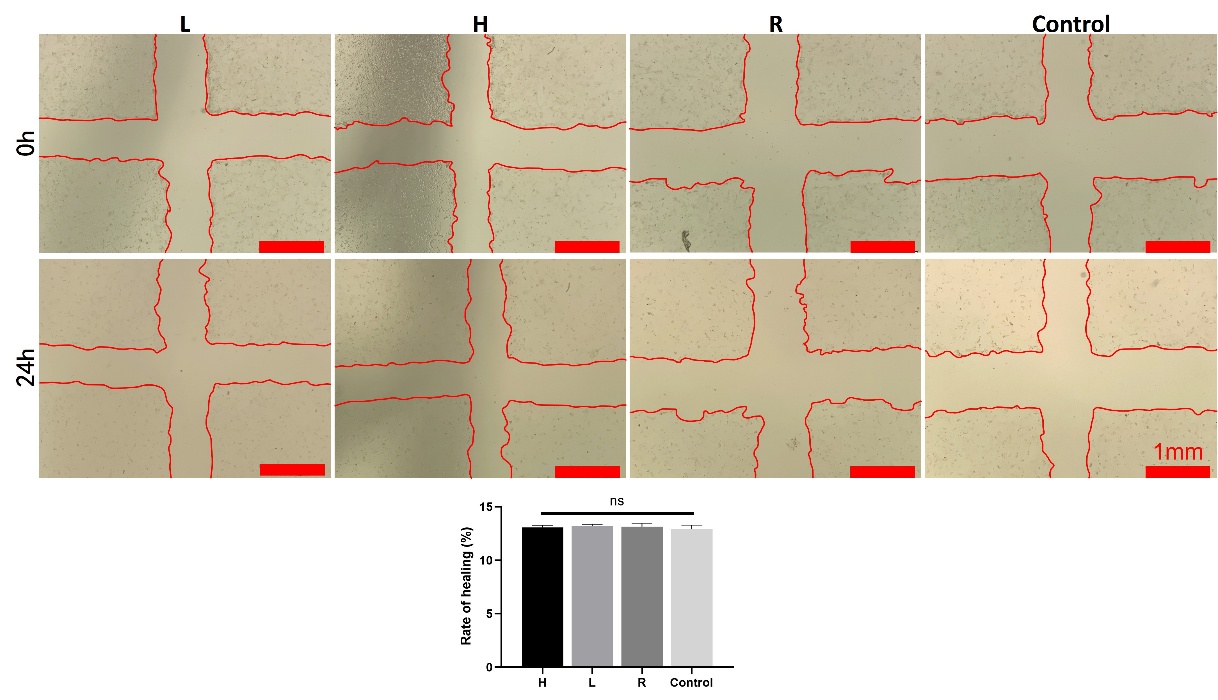


Fig. S2 The Images of the wound healing assay conducted using the matrix scaffold extract and the analysis of its healing rate


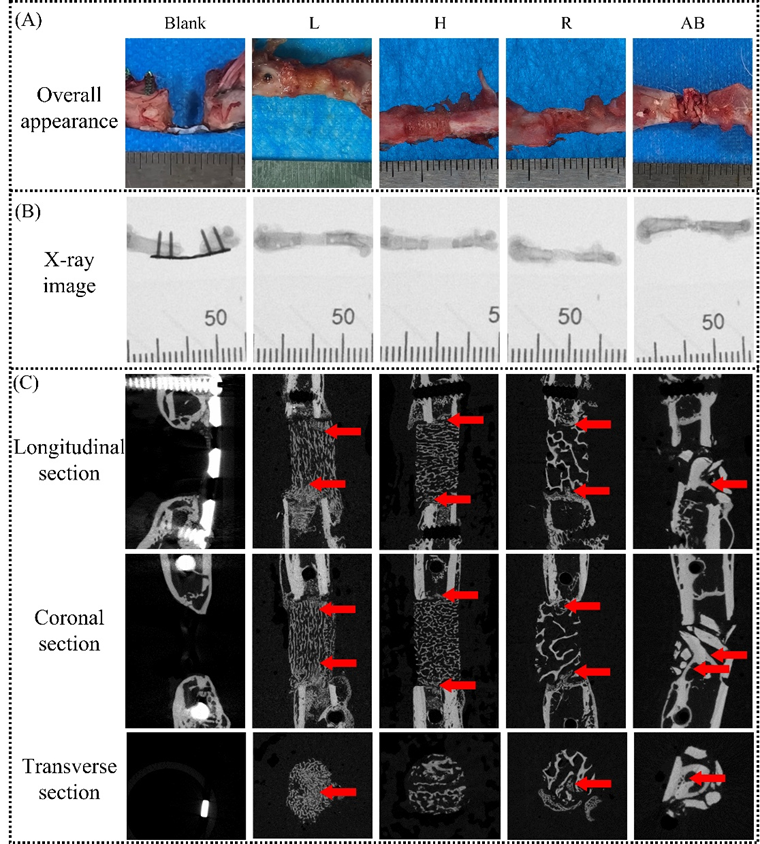


Fig. S3. Imaging manifestations of in situ implantation experiments. (A)The overall appearance of rat femur after implantation of four weeks. (B)X-ray image of rat femur after implantation of four weeks. (C) Micro-CT trisection of rat femur after implantation of four weeks (red arrow: newborn bone).
